# Rheological characterisation of synthetic and fresh faeces to inform on solids management strategies for non-sewered sanitation systems

**DOI:** 10.1016/j.jenvman.2021.113730

**Published:** 2021-12-15

**Authors:** Edwina Mercer, Shane P. Usher, Ewan J. McAdam, Brian Stoner, Yadira Bajón-Fernández

**Affiliations:** a School of Water, Energy and Environment, Cranfield University, Bedfordshire, MK43 0AL, UK; b Department of Chemical Engineering, The University of Melbourne, 3010, Australia; c Centre for WASH-AID, Duke University, Durham, NC, 27708, United States

**Keywords:** Yield stress, Gel point, Sticky phase, Dewatering, Drying, Faeces

## Abstract

In order to obviate the economic issues associated with pit latrine emptying and transport such as high water additions and rheologically difficult sludge properties, the implications of prompt solid/liquid separation were investigated. This was achieved through rheological characterisation of fresh human faeces and synthetic faeces, and comparison with aged faecal sludges. Shear yield stress, thixotropy and post-shear structural recovery were characterised for a total solids (TS) concentration range of 5–35% total solids (TS) and stickiness yield stress was determined for concentrations up to 100% TS. Fresh faeces rheology proved to be favourable when compared to aged matrices, evidenced by a lower shear yield stress and higher gel point solids concentration, suggesting that aging could alter the physico-chemical properties of faecal sludge. Fresh and synthetic faeces exhibited similar shear thinning, thixotropic behaviour with the majority of structural breakdown occurring at a low shear rate of 10 s^−1^, and the extent increasing with higher solids concentrations. At 32% TS, fresh faeces shear yield stress was permanently reduced by 80%, suggesting that low shear pumping could reduce the energy demand required for faeces transport. The sticky phase, which represents the region to avoid faecal transport and mechanical drying processes, was identified to range from 30 to 50% TS, with 25% TS as ideal to commence dewatering processes. This also coincides with the average solids concentration of faeces, which is achievable by source separation. This study has identified that handling of fresh faeces as opposed to aged faecal sludges would result in economic and environmental benefits, with energy, water and labour savings.

## Highlights

• Early intervention is favourable due to lower shear yield stress and higher gel point.
• Fresh faeces shear yield stress can be reduced by low shear structural deformation.
• Sticky phase was at 30-50% TS, where transport and mechanical drying should be avoided.
• Early intervention can reduce pump head, energy and need for water addition.
• Synthetic faeces had similar rheological behaviour to real faeces.

## Graphical abstract

**Figure:**
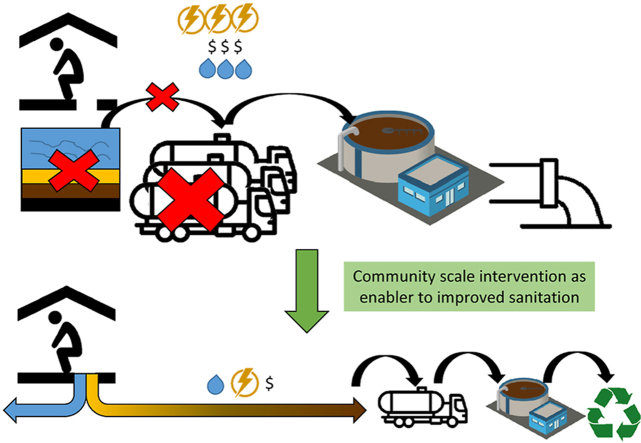

## 1 Introduction

The United Nations (UN) estimates that 61% of the global population lack safely managed sanitation and 29% lack clean water services (United Nations, 2018). In light of this, the UN have actioned the Sustainable Development Goal 6 (SDG 6) aiming to provide global access to sanitation and clean water by 2030. However, in low-income countries (LICs), a conventional centralised wastewater treatment infrastructure where sewerage contributes to 84% of capital costs (Jung et al., 2018), is economically unfeasible. Consequently, pit latrines are a ubiquitous intermediate solution to decentralised sanitation providing localised sludge storage, which eventually require emptying, transport and treatment, as often as every 13 months (Chidya et al., 2016; Cole et al., 2012). Pit emptying fees range from $35 - $95 per household in India and Kenya respectively, representing a substantive portion of an average monthly salary ranging from 12 to 125% (Chowdhry and Kone, 2012; World Bank, 2019). Furthermore, due to the long storage periods, pit latrine sludge is stratified, with the fresher liquid layers towards the top and degraded consolidated sludge towards the bottom, reported to have densities of up to 1750 kg m^−3^ (Radford and Sugden, 2014) where a considerable amount of added water is required to reduce sludge viscosity to facilitate a sufficient drop in pump head and power requirements (Septien et al., 2018a; Strande et al., 2014). This practice is, however, restricted by pit volume and water availability and incurs longer emptying times and greater transport costs which constitute 25% of faecal sludge management costs (Steiner et al., 2002). In the dense urban setting, where limited space prevents the use of large capacity sludge tankers, intermediate small-scale vehicles restricted by volume (up to 1000 kg) and distance (up to 3 km) alternatively take sludge to a fixed transfer station to be collected by larger tankers (Mikhael et al., 2014). Silting and layering reoccurs during storage, resulting in further difficulties in sludge transfer and extra costs are incurred before reaching treatment (Mikhael et al., 2014). Current pit latrine emptying and transport practices can therefore become costly due to ineffective solid liquid separation. As a consequence, it is reported that only 22% of pit latrine sludge is safely managed in the urban setting (Blackett et al., 2014). In Dhaka, Bangladesh, only 0.3% of faecal sludge reaches treatment with residents preferring to manually empty hazardous faecal sludge directly into the surrounding drains in order to avoid emptying and transport costs (Ross et al., 2016).

In order to progress faecal sludge management toward the SDG6 aspirations, the technical feasibility of sludge transport must be improved. Focus should shift towards reducing transportation volume which is linked to the rheological properties which govern both the flowability and dewaterability of the suspension. This ultimately determines transportation costs for collection. Community based intervention strategies present one such approach where advanced solid/liquid separation could be introduced to target the fresher unconsolidated faecal sludge. Such units could recover faecal solids to a dry friable form which in turn reduces volume, thereby improving the economics of transport as well as reducing pathogenic risk (Brockmann, 1973; Naidoo et al., 2020). Furthermore, this concentrated faecal sludge fraction comprises solely of blackwater constituents rich in nutrients and organics with direct opportunity for nutrient or energy recovery, encouraging local economic opportunities for sustainable sanitation (Eshetu Moges et al., 2018; Forbis-Stokes et al., 2016; Harder et al., 2019; Onabanjo et al., 2016). As such, localised solid/liquid separation could enable a community accepted shift towards sustainable faecal sludge management (Mikhael et al., 2014).

Characterisation of the rheological behaviour of faecal sludge is critical to inform on both the design and operation of dewatering or drying processes. Solids concentration is considered to be the defining factor which influences the rheology of faecal sludge (sewage sludge, pit latrine sludge, blackwater and faeces) by proportionally inducing shear stress to yield flow (Cao et al., 2016; Septien et al., 2018a; Thota Radhakrishnan et al., 2018a; Thota Radhakrishnan et al., 2018b; Woolley et al., 2014b). However, faecal sludge rheological values differ according to type and literature on fresher unconsolidated faecal material is limited. Woolley et al. (2014a, 2014b) provided an insight into the rheological behaviour of fresh faeces, reporting shear thinning and thixotropic properties. Thota Radhakrishnan et al., 2018b, rheologically characterised blackwater comprising urine, faeces and flush water in a vacuum toilet. However, standing time was not reported, which is key to determining the impact of faecal aging on rheology from changes in physico-chemical properties. In addition, these studies utilised a bob and cup fitting technique, which was the predominantly used technique for faecal sludge characterisation to date (Eshtiaghi et al., 2013; Thota Radhakrishnan et al., 2018a; Thota Radhakrishnan et al., 2018b; Woolley et al., 2014a, 2014b). We suggest replacing the conventional bob and cup fitting with vane geometry for rotational rheometry techniques of faecal material as it provides substantial clearance between the vane and the container wall, avoiding the lodging of large particles (Fisher et al., 2007) and allows yielding to occur between layers rather than against the cup wall, minimising the effects of wall slip (Barnes and Carnali, 1990). Faeces is a heterogeneous particulate material containing large un-masticated food pieces such as seeds, corn and tomato skin (Mercer et al., 2016). Insertion of the vane causes minimal disturbance, which is critical for a thixotropic material such as faeces, to characterise shear yield stress (τ_y_), which has only been inferred by extrapolation using the bob and cup geometry (Thota Radhakrishnan et al., 2018a; Thota Radhakrishnan et al., 2018b). Yield stress is essential for identifying the minimum energy requirement to initiate flow. Through detailed investigation of thixotropic structural deformation, it can inform the extent of improved flowability of the suspension. Faecal sludge also possesses adhesive properties attributed to solids concentration which correspond to regions requiring high stress to initiate sliding, described as the ‘sticky phase’ (Peeters et al., 2011). Beyond 25% TS, faeces becomes a semi-solid material which is unable to flow, presenting a limit for conventional rheological characterisation (Septien et al., 2018a). The rheology of dewatered and dried faecal material is therefore unrepresented, which is equally required considering the importance of solids concentration. Through investigating stickiness yield stress (τ_s_) across a broad solids concentration range, we can determine transport behaviour when faecal material no longer yields by flow, but by sliding. This study therefore introduces a second new methodological approach for the characterisation of faecal sludge. Peeters et al. (2011) were able to identify the τ_s_ of activated sludge using a simple lab protocol for stickiness based on the mass required to initiate sliding of a material of a determined surface area, along a steel surface. As a result, the sticky phase could inform on sludge management strategies.

This study seeks to advance knowledge on the rheological characterisation of fresh human faeces in order to inform on the potential of alternative community based interventional strategies that can improve faecal sludge management. In doing so, methodologies novel to the study of fresh human faeces (vane geometry, sticky phase) are introduced to permit rheological characterisation of fresh faeces across a broad solids concentration range. Conditions at source (5% TS) through to materials that have been subject to more advanced solid-liquid separation techniques such as dewatering and drying (100% TS) have been represented. The specific objectives are to: (a) quantify the yield stress of fresh human faeces; (b) benchmark yield stress data versus a broader suite of fluids to interrogate the significance and implications of the data; (c) characterise the thixotropy, structural recovery and establish the rheological model for fresh human faeces; and (d) identify the sticky phase region for fresh human faeces to inform on the suitability and implementability for technical solutions. Whilst significant sanitation research is ongoing, progress has been slowed by access to fresh human faeces due to ethical concerns, practical limitations (e.g. unsuitable laboratory pathogenic risk management) or the limitation to donations, such that synthetic faeces recipes have been established and widely adopted (Penn et al., 2018). Therefore, the final objective of this study is to characterise the rheological properties of synthetic faeces and compare this to real human faeces to determine their suitability as a proxy.

## 2 Materials and methods

### 2.1 Preparation and collection of synthetic and fresh faeces

Synthetic faeces (SF) developed by Penn et al. (2018) was identified to be rheologically comparable to real faeces (RF), however limited to one condition (apparent viscosity at 50 rpm at 20% TS). This study thoroughly characterises the rheological properties of this synthetic substitute using the same methods as with RF for a validated comparison (yield stress, shear rate response curves, thixotropy and structural recovery), and extends to a range of solids concentrations typical for source separated sanitation systems (5–35% TS). The recipe SE65 (Table 1) was prepared at 35% TS to be used as the stock material (Penn et al., 2018).

**Table 1:** Synthetic faeces recipe SE65 at 35% total solids, adapted from Penn et al. (2018).

Ingredient	Mass for 1 kg of synthetic faeces (g)
Yeast extract	127
Microcrystalline cellulose	42
Psyllium	74
Miso paste	74
Peanut oil (substitute for oleic acid)	84
NaCl	8
KCl	8
CaCl_2_.H_2_0	5
Deionised water	578

Real samples were obtained from consenting anonymous volunteers through a collection regime approved by the Cranfield University Research Ethics System (CURES, project ID 8488). Seventy-five faeces were collected for rheological and sticky phase characterisation, classified by the Bristol Stool Scale (BSS) according to Heaton et al. (1992a) (Table 2) and their TS concentration determined using standard methods (APHA, 2012).

**Table 2:** Stool types as described by Heaton et al. (1992a).

Bristol Stool Scale (BSS)	Description
BSS 1	Hard dense lumps
BSS 2	Lumpy sausage
BSS 3	Cracked sausage
BSS 4	Smooth sausage
BSS 5	Soft defined blobs
BSS 6	Mushy stool
BSS 7	Watery, no solid pieces

Both SF and RF were refrigerated in closed containers for less than a week to maintain freshness. All samples were analysed at room temperature (20 °C) and each experiment was repeated in triplicate on sub-samples and verified as replicable. For rheological characterisation, stored samples of known solids concentration were diluted with deionised water, homogenised (100 s^−1^ for 30 s using a vane) and left to stand to allow for structural recovery for 24 h. The gel point (ϕ_g_), defined as the solids concentration where particles form a networked structure, was determined from batch settling tests as the average TS at the final sedimentation height (Lester et al., 2005). Samples below ϕ_g_ (<10% TS) were re-suspended (10 s^−1^ for 10 s using a vane) immediately before an experiment according to standard protocol of addressing rapid settling slurries (Akroyd and Nguyen, 2003), in order to maintain a consistent solids concentration throughout the beaker due to the rapid sedimentation of faecal particles. Initial testing identified that resuspension occurs from 5 s^−1^. For the τ_s_ tests, faeces samples were also diluted accordingly or empirically dried in an oven at 105 °C to cover a solids concentrations range of 5–99% TS (Peeters et al., 2011).

### 2.2 Rotational rheometry techniques

A Haake Viscotester iQ rheometer with vane rotors (Thermo Electron, Karlsruhe, Germany) was utilised to characterise the rheological properties of SF and RF. A large FL40 4B/SS vane (H = 55 mm, D = 40 mm) was used to provide resolution for the low torque responses encountered in the diluted samples (≤15% TS). Conversely, a smaller FL22 4B/SS vane (H = 16 mm, D = 22 mm) was utilised to remain within the measurement limits of the high torque responses for the undiluted samples (>15% TS). The vanes were immersed in a container at least twice the height and diameter of the vanes (H = 130 mm, D = 80 mm and H = 70 mm, for FL 40 4B/SS and H = 130 mm, D = 50 mm and H = 70 mm for FL22 4B/SS) as advised by Nguyen and Boger (1985) for concentrated suspensions. Such gap widths were at least 5 times larger than the observed unmasticated food particles encountered in faeces. Haake RheoWin Data Manager converts angular velocity and torque from the vane rotation to shear rate, shear stress and viscosity.

The vane rotated according to the program on Haake RheoWin Job Manager ‘Yield stress determination using a vane rotor’. This method sets a low rotational speed of 0.05 rpm at the vane radius, which was maintained for 300 s (Fig. S1). After an initial purely elastic response in the sample, the structure fails and the shear stress decreases again. The maximum shear stress value then corresponds to the τ_y_ (Nguyen and Boger, 1985). Shear yield stresses of RF and SF were also compared to other shear thinning faecal sludge matrices such as aged faeces and wastewater (Septien et al., 2018a; Trávníček and Junga, 2014), which also used the vane method for quantification. Aged faeces values were derived from the r^2^ line equation expressing τ_y_ as a function of solids concentration (r^2^ = 0.9408, τ_y_ = 0.2404e^0.3598∙TS^) reported in Septien et al. (2018a). Wastewater sludge values were acquired from the sludge rheology database software (Slot 2.0, BHR, 2020), representing primary unthickened sludge (1% and 5% TS), primary thickened sludge (10% TS) and secondary activated sludge (12% and 19% TS).

The shear rate was doubled incrementally from 0.001 to 100 s^−1^ at 20 s per step with the shear stress and viscosity response recorded in order to characterise the faeces rheological model, which was overlaid by RheoWin Data Manager Software. The shear rate was also ramped down at the same rate to exhibit the thixotropic nature of faeces.

Structural recovery was conducted using the standard three phase ‘Structural recovery’ protocol according to Haake RheoWin Job Manager. A pre-shear period of 30 s was introduced at 0.05 rpm (τ_y_ shear rate) to allow for the yield stress to be reached, followed by a 10 s shear period at 10 s^−1^ which initiated structural deformation. A shear rate of 10 s^−1^ was identified as the region in which most structural breakdown occurred during thixotropic characterisation. A 360 s post-shear period (0.05 rpm) was then introduced to allow for structural recovery, indicated by a plateau in shear stress, (Fig. S2). The extent of recovery was determined by the shear stress percentage difference between the pre and post-shear periods. Sample yield stress values were measured again 24 h later to assess whether any further recovery had occurred, in which the same post shear recovery values were observed.

### 2.3 Determination of the stickiness of fresh faeces

The τ_s_ of wastewater sludge was previously characterised and validated by Peeters et al. (2011) in order to determine the ‘sticky phase’ of activated sludge, informing when to transport sludge during dewatering and drying processes. Stickiness yield stress is defined as the mass required for a material to overcome adherence to a stainless steel surface. A rig was adapted from Peeters et al. (2011) to similarly characterise the ‘sticky phase’ of faeces. It comprises an open cylinder (H = 10 cm, ID = 5.5 cm) containing 15 g of consolidated faeces which slides along a stainless steel surface. Faeces are consolidated with a weighted cylinder (W = 2.2 kg, OD = 5.3 cm) which fits inside the open cylinder, for 1 min. It is recommended to coat the cylinder in parafilm to prevent faeces sticking when removed. Sliding is initiated by incrementally adding mass (water or weights) to a bucket, which is attached to the open cylinder via a steel wire and weight redirected to vertical axis by a pulley. The bucket is weighed when the open cylinder starts to slide. Stickiness yield stress is calculated as:

(1) $$\;\tau_{s}=\;\frac{m\times g\;}{A_{c}}$$

where τs is the stickiness yield stress (Pa), m is mass of the filled bucket (kg), g is gravity (9.81 m s^−2^) and A_c_ is the open cylinder contact area (m^2^).

## 3 Results and discussion

### 3.1 Early intervention of faeces reduces its shear yield stress

**Fig. 1:**
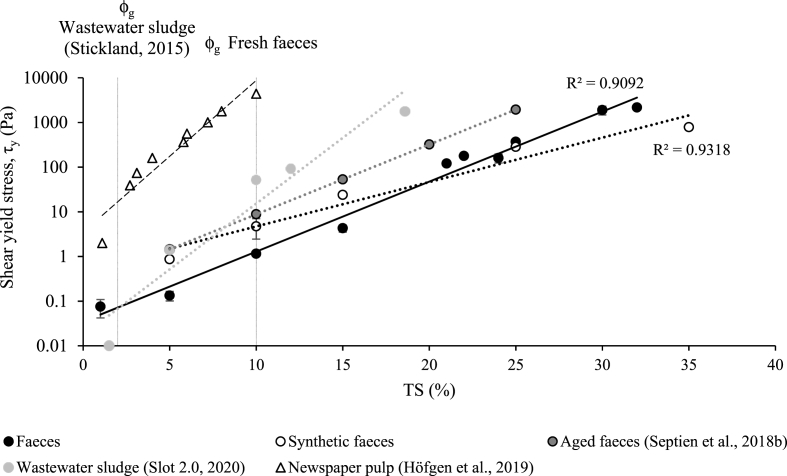
Comparison of synthetic faeces, real fresh faeces, real aged faeces, wastewater sludge and newspaper pulp shear yield stress (τ_y_) as a function of total solids (% TS), overlaid with the gel point (φ_g_) of fresh faeces and wastewater sludge. Data obtained using vane test (Nguyen and Boger, 1985) at 0.05 rpm for 300 s on samples at 20 °C. Error bars represent the standard deviation of triplicate subsamples.

Shear yield stress was investigated to provide values of the minimum energy requirements for faeces to flow from stasis (Fig. 1). Shear yield stress increases exponentially for both SF and RF with both materials exhibiting a strong correlation (r^2^) of 0.9577 and 0.9859, respectively, with TS content. However, RF possess a steeper gradient at τ_y_ = 0.0348e^0.3608∙TS^ compared to τ_y_ = 0.4755e^0.229∙TS^ for SF. When diluted, SF exhibit a higher yield stress than RF and a crossover exists when RF are undiluted at ∼25 %TS. When comparing τ_y_ with other faecal matrices, aged faeces (AF) acquired from pit latrine sludge (Septien et al., 2018a) presented τ_y_ values higher than fresh faeces by an order of magnitude (τ_y_ = 0.2404e^0.3598^^∙TS^). Wastewater sludge (Slot 2.0, BHR, 2020) provided a steeper positive gradient response than AF and RF (τ_y_ = 0.0174e^0.6775^^∙TS^) which ultimately led to respective τ_y_ responses of 1764, 500 and 120 Pa for wastewater, AF and RF at ∼ 20% TS. Wastewater and AF have differing degrees of digestion (∼50% VS and ∼65% VS, respectively) and dilution from external substrates such as trash, greywater and rainwater (Septien et al., 2018a). Wastewater sludges typically have a gel point at 1–2% v/v (Stickland, 2015), compared to 10% w/v of RF and SF. The ϕ_g_ is an indicator of dewaterability of compressible suspensions at low solids concentrations, due to the fact that there is an inherent network strength at solids concentrations greater than ϕ_g_ that requires a compressive mechanical stress to dewater and a critical shear stress to yield and flow. At solids concentrations lower than φ_g,_ passive sedimentation is sufficient for dewatering and τ_y_ is negligible. In wastewater and AF, poor dewaterability is associated with extracellular polymeric substances (EPS), where organic content can also be used as an indicator (Skinner et al., 2015). Such substances form networked floc structures which are highly compressible, translating to a substantial energy demand and processing time to reach high solids concentrations. Such fortified structures would also require additional energy to initiate flow. In fresh faeces, organic content exceeds that of wastewater sludge (≥80% volatile solids, VS) consisting of bacterial biomass with undigested proteins, carbohydrates, fibres and fat (Rose et al., 2015). However, these components behave as particles having recently undergone effective compression and dehydration by the colon (Lewis and Heaton, 1997). It is therefore hypothesised that when fresh, the microbial biomass which makes up ∼50% of faeces (Rose et al., 2015), have not had the opportunity to secrete compressible and impermeable EPS floc structures. We hypothesise that the structure and physico-chemical properties of faeces change with degradation during storage, consequently influencing dewaterability and rheological behaviour. This confirms the importance of practicing early solid/liquid separation which is not only beneficial to avoid reported sludge compaction issues, but to maintain the physical properties which are advantageous: lower τ_y_ at the same TS% of aged faecal matrices and a higher ϕ_g_ concentration reached. The emptying and tankering of aged sludge is therefore counterintuitive for cost effective faecal sludge management. The dataset presented is directly relevant to contexts where water is used for anal cleansing. Further research is required to elucidate the impact of matrix properties, including fiber type and content, in order to fully inform sanitation economics when a variety of papers are used for cleansing. Höfgen et al. (2019) investigated the compressional and shear rheological characteristics of newspaper pulp, which was identified as a highly permeable material. However, the rigid fibres inhibit the compressional effects of gravity, causing a gel point solids concentration of 0.011 v/v (∼1.4% TS), compared to 0.084 v/v (∼10% TS) of faeces. Similarly, shear yield stress is impacted by the strength of the paper fibres, with the shear yield stress of newspaper pulp being three orders of magnitude greater than fresh faeces (Fig. 1). Therefore, it is initially anticipated that benefits will result from separating paper used for cleaning purposes, although further research is required to fully understand the rheological characteristics of faeces and paper as a composite material.

### 3.2 Further reduction of yield stress achievable by inducing low shear to facilitate structural deformation

**Fig. 2:**
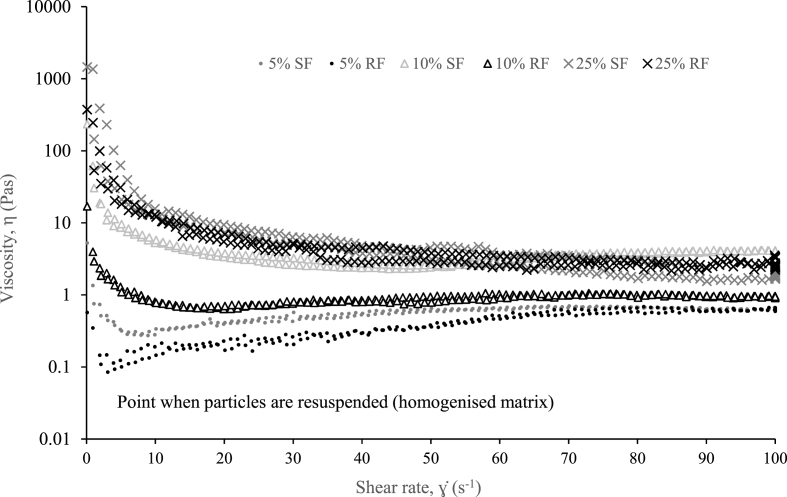
Comparison of synthetic faeces (SF) and real faeces (RF) apparent viscosity as a function of shear rate at solids concentration below (5% TS), at (10% TS) and above (25% TS) the gel point (φ_g_).

At solids concentrations lower than ϕ_g_ (such as 5% TS), an initial higher apparent viscosity with shear rate is observed for RF and SF caused by a higher solids concentration settled at the base of the vane (Fig. 2), which decreases with homogenisation. A homogenised mixture occurs at rotational speeds greater than 5 s^−1^ in which apparent viscosity remains constant (Newtonian-like behaviour) and is within the same order of magnitude for both RF and SF. At 10% TS (φ_g_) and above the solids concentration representing ϕ_g_ (25% TS), RF and SF are shear thinning (Fig. 2), which is in line with Woolley et al. (2014b). At 10% TS, the same behaviour exists between RF and SF, however differentiated by an order of magnitude. At 25% TS (Fig. 2), the viscosity of SF matches that of RF at the shear rates between 0.001 and 100 s^−1^ and can therefore be classed as an accurate substitute of faeces at its average solids concentration (Rose et al., 2015).

**Fig. 3:**
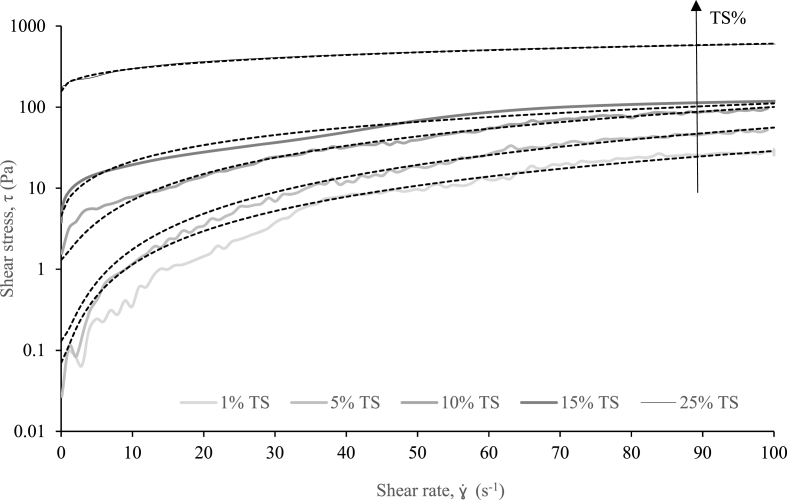
(a) Shear rate vs. shear stress curves for fresh faeces at varying solids concentrations (% TS, total solids). Overlaid with rheological models (Table 3).

Real faeces generally follow a Herschel-Bulkley rheological model (Fig. 3 and Table 3) as reported by Woolley et al. (2014a, 2014b) for fresh faeces at higher solids concentrations, and other faecal sludge matrices such as wastewater and AF (Septien et al, 2018a, 2018b, 2018a; Trávníček and Junga, 2014). At solids concentrations lower than ϕ_g_ (<10% TS), τ_y_ is below 1 Pa as particles flow within a liquid medium, therefore the model can be simplified to a power law model. At solids concentrations higher than ϕ_g,_ τ_y_ becomes significant and the networked structure requires increased stress to flow. The ϕ_g_ has evidenced its influence on the rheological behaviour of both RF and SF, providing the transition between Newtonian and shear thinning (Fig. 2) and defining the limit when τ_y_ exists (Table 3). It is a parameter which has never been investigated before for RF or AF.

**Table 3:** Faeces rheological model.

Total solids (%)	Model equation	Correlation r	Yield stress $\tau_{y}$	Parameter K	Parameter n
1	$\tau =\tau_{y}+K{\dot{\gamma }}^{n}$	0.9907	0.07	0.04002	1.428
5		0.9949	0.13	0.04747	1.535
10		0.9948	1.3	0.3423	1.231
15		0.9913	4.569	2.682	0.7996
25		0.9990	156.3	44.04	0.5050

**Fig. 4:**
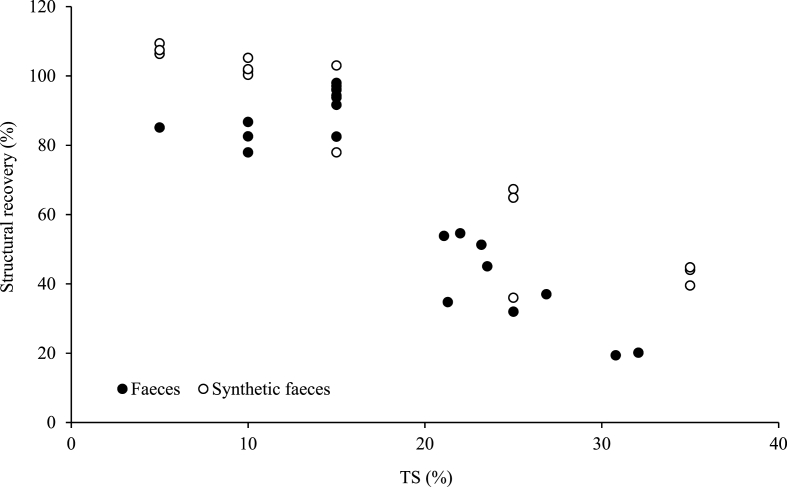
Structural recovery of shear yield stress of real and synthetic faeces. 30 s pre-shear (0.05 rpm), 10 s shear (10 s^−1^), and 360 s post-shear (0.05 rpm). Data points represent individual samples.

Structural recovery experiments allowed for the quantification of shear induced (10 s^−1^ for 10 s) structural deformation (Fig. 4). For the diluted RF and SF samples, recovery from 80 to 100% could be reached after 30 s recovery time. For the undiluted samples, the extent of recovery decreases with solids concentration (r^2^ = 0.7063, y = −2.634x + 103.79) from 32% at 25% TS to 20% at 32% TS. Synthetic faeces experienced a lower extent of structural breakdown of 65% at 25% TS and 40% at 35% TS. Undiluted samples required 300 s to reach a constant recovery value. These samples were also checked 24 h later to assess any τ_y_ change, in which no further recovery was observed. The extent of structural recovery after shear for SF is double that of RF, and therefore overestimates the energy requirements for dynamic pumping processes if used as a surrogate material. The prospect of permanent structural deformation highlights that although faeces possess higher shear stresses at higher solids concentrations, they can be managed by a short period of low shear homogenisation. This provides a consistent matrix and a τ_y_ reduction of up to 80%.

**Fig. 5:**
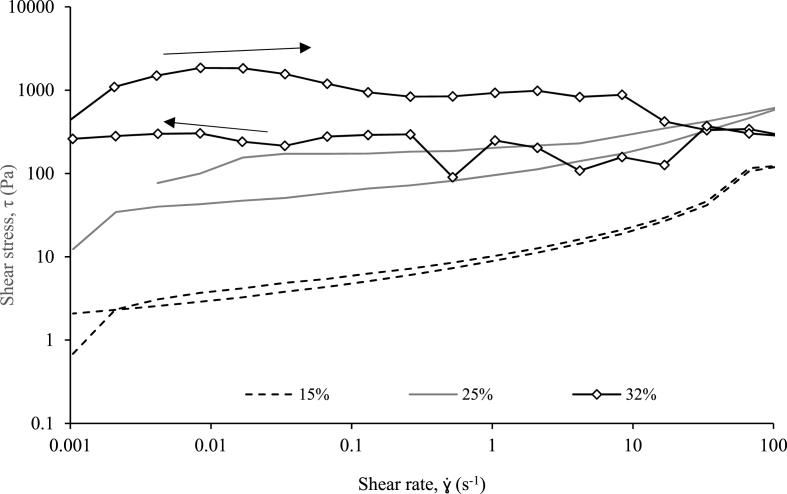
Comparison of shear stress versus shear rate hysteresis at solids concentrations above the gel point (15%, 25% & 32% total solids) after ramping shear rate up at down.

An increase in thixotropic behaviour can be observed with increasing solids concentrations through increased structural breakdown as with the structural recovery tests (Fig. 4, Fig. 5), although with greater resolution on the impact of shear rate (0.001–100 s^−1^). However, it is unclear whether the structural deformation exhibited by the samples >25% TS is purely a result of thixotropic breakdown. The 32% TS sample is characterised by an uneven shear rate ramp line, in addition to constant shear stress across all shear rates, which suggests that the material is no longer flowing and fracturing (Tanner and Keentok, 1983) or segregation (Hoang et al., 2015) phenomena could be occurring in addition to thixotropic breakdown. Such behaviour is analogous to other semi-solid materials such as bread dough, when encountering high shear rates (Tanaka, 2012; van Vliet, 2013). The other undiluted sample (25% TS), demonstrates flowing behaviour with the smooth ramp line in addition to increasing shear stress response to shear rate, and can therefore be considered as thixotropic breakdown. Beyond 25% TS can therefore be regarded as the TS limit for rheological characterisation of fresh faeces as reported by Septien et al. (2018a) for AF. Importantly, it can be observed that beyond a shear rate of 10 s^−1^, the extent of thixotropic breakdown is minimal confirming that increasing the homogenisation shear rate would not provide greater deformation, therefore identifying the limit for energy efficient homogenisation.

### 3.3 Avoid faecal transport and mechanical drying processes between 30 and 50% TS

**Fig. 6:**
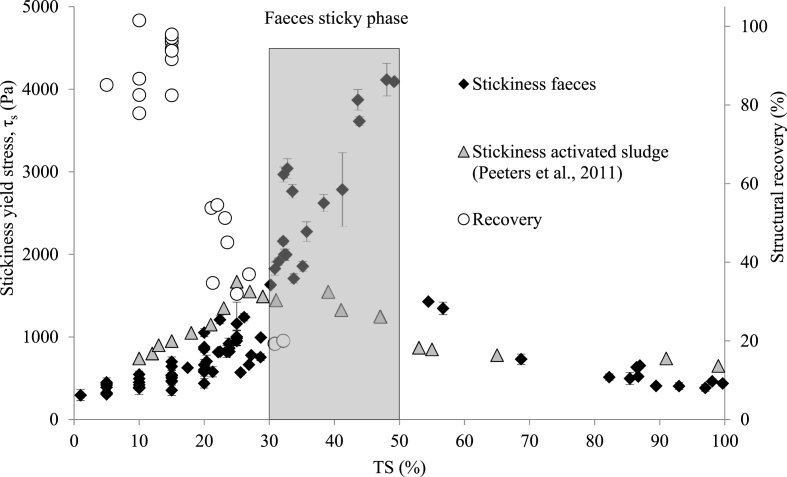
Stickiness yield stress as a function of total solids (% TS) for fresh faeces, overlaid with wastewater activated sludge and shear yield stress structural recovery (Fig. 4). Error bars represent the standard deviation from triplicated subsamples.

Stickiness yield stress also plays an important role in the design of solid phase processing, particularly towards the drying end of the solids phase process spectrum. Wastewater sludge goes through an adhesive phase in which it sticks to surfaces resulting in reduced transport efficiency and operational problems (2011). Below 30% TS (Fig. 6), RF solids concentration is moderately linked to the extent of stickiness (r^2^ = 0.67, y = 26.988x + 185.66). From 30% TS τ_s_ increases linearly until reaching 50% TS (r^2^ = 0.75, y = 120.36x - 1768.2), at which a sudden reduction of adherence to surfaces is observed by a factor of 2.5 (y = −21.766x + 2464). When comparing with activated sludge (2011), fresh faeces requires twice the force of activated sludge stress to overcome adherence to steel surfaces within the sticky phase identified as 15–50% TS and 30–50% TS for activated sludge and fresh faeces respectively (Fig. 6). This highlights the variability between faecal sludge types, and the importance of characterising τ_s_ across a range of faecal sludges. For fresh faeces, it is paramount to avoid transport and mechanical drying processes when stickiest between 30 and 50% TS, potentially reducing energy demand by almost threefold. Instead, emphasis should be on dewatering to exceed 50% TS or alternative non-mechanical drying processes such as drying beds (Strande et al., 2014).

### 3.4 Faecal rheological behaviour can be predicted by solids concentration

**Fig. 7:**
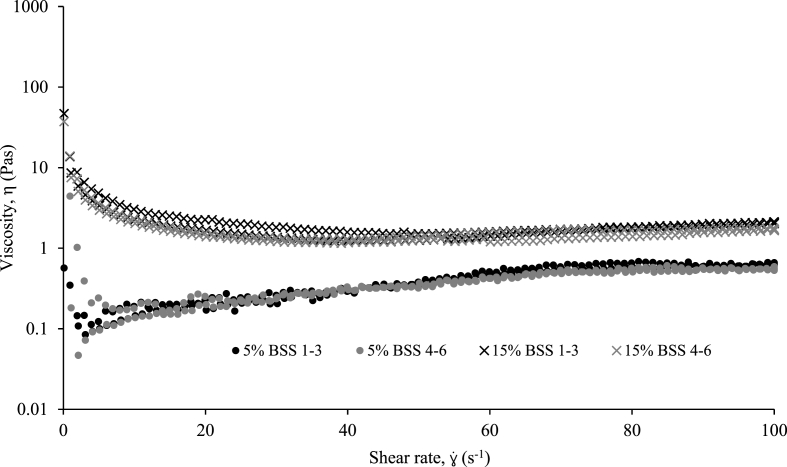
Comparison of BSS 1–3 and 4–6 apparent viscosity response as a function of shear rate ramped up and down, below (5% total solids) and above (15% total solids) the gel point solids concentration.

**Fig. 8:**
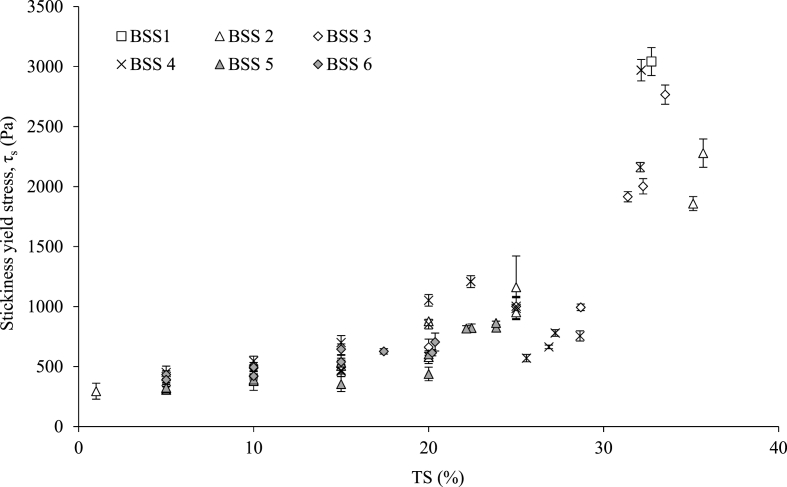
Comparison of stool types (BSS) stickiness yield stress according to total solids (TS, %). Error bars represent the standard deviation of triplicated subsamples.

Faeces come in different forms as categorised by Heaton et al. (1992b), through the BSS described in Table 2, where form is attributed to intestinal transit time and therefore increased faecal dehydration with time (Lewis and Heaton, 1997). Particularly, there are distinct differences between stool types 1–3 and 4–6 in terms of solids concentration (Fig. S3), where BSS 1–3 and 4–6, possess median solids concentrations of greater than 30% TS and less than 25% TS, respectively. In order to identify any further distinguishing factors, shear rheology (Fig. 7) and τ_s_ (Fig. 8) were examined in higher resolution according to stool type. When assessing the shear vs. viscosity response above and below the ϕ_g_ solids concentration, identical curves were presented for all BSS faeces types (Fig. 7). Similarly, τ_s_ was primarily influenced by the solids concentration attributed to the stool type (Fig. 8). Overall, there is no distinct difference between stool type other than by solids concentration, which allows for the prediction of rheological behaviour and an unbiased comparison with SF.

### 3.5 Environmental benefits of early intervention

**Fig. 9:**
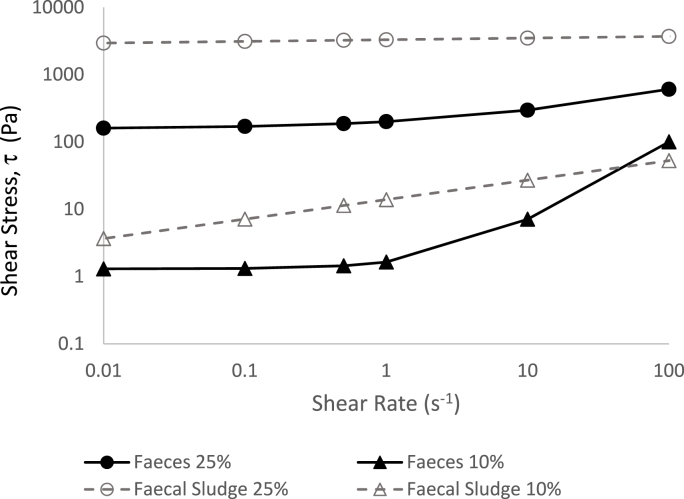
Comparison of rheological curve fits of fresh faeces (this study) and aged faecal sludges (data from Septien et al. (2018a)) at 10% and 25% total solids concentrations. The rheogram (shear stress vs shear rate) was obtained with a Herschel Bulkley model fitting for fresh faeces (this study) and with a power law model for aged faecal sludge (from Septien et al. (2018a)).

The positive environmental and economic impacts of transporting fresh faeces as opposed to aged matrices were estimated by quantifying the power, water and time required to empty a pit latrine of 1 m^3^. Rheological behaviour for a range of shear rates was compared for fresh faeces (data of this study) and aged faecal material (data from Septien et al. (2018a)) at 10% and 25% TS (Fig. 9). At a nominal shear rate of 0.5 s^−1^, the faecal sludge has a shear stress 7.9 and 17.3 times higher than the fresh faeces for 10% and 25% TS, respectively (Fig. 9). This translates to about ten times the pumping power requirement at the same flowrate and solids concentration when handling aged faecal sludges.

For comparative purposes, a similar exercise as that reported by Septien et al. (2018a) was conducted, calculating the additional water required to be mixed with aged faecal sludges to reduce hydraulic head to levels estimated for fresh faeces. It was estimated that for a 1 m^3^ initial volume of 25% TS faecal sludge (Septien et al., 2018a) to have a rheology comparable to fresh faeces at 0.5 s^−1^, dilution to 17.6% TS would be required using an additional 445 L of dilution water. Similarly, for a comparable rheology with 10% TS faeces at 0.5 s^−1^, 1000 L of faecal sludge would need to be diluted to 4.4% TS using an additional 1300 L of dilution water. Clean water costs between £0.0014–0.0368 per L (USD$ 0.002–0.05) in the UK and Papua New Guinea respectively (WaterAid, 2016), which equates up to USD$65 for 1300 L of dilution water. This water addition cost can constitute a significant fraction of pit latrine emptying costs (Chowdhry and Kone, 2012). These dilutions would also translate to increases in pumping time ranging from 45 to 130% for faecal sludge relative to fresh faeces, increasing associated labour costs and energy requirements in addition to the environmental impact of sourcing, transporting and treating the extra dilution water. The significant potential savings in energy and water make a compelling case for seeking to handle fresh faeces in preference to aged faecal sludges.

## 4 Conclusions

This study has demonstrated that early intervention solid/liquid separation is thermodynamically favourable for faecal solids management. Fresh faeces require a lower shear stress to initiate flow, thereby reducing pumping head. At 20% TS, τ_y_ values are 1764, 500 and 120 Pa for wastewater, aged and fresh faeces respectively, which suggests that physico-chemical changes occur with older faecal matrices. This is supported by the φ_g_ of fresh faeces identified at 10% TS, which is five times greater than wastewater sludge (Stickland et al., 2008), without the addition of polymer. For fresh faeces, the existence of shear yield stress corresponds to the formation of a fortified networked structure (φ_g_)_,_ demonstrating an opportunity for low energy pumping from source to treatment (τ_y_ ∼ 0.1 Pa at concentrations lower than φ_g_). A non-zero compressive yield stress also becomes apparent at solids concentrations greater than φ_g_ and provides an indication of fresh faeces dewaterability at low solids concentrations (diluted faecal matrices) by identifying the extent of solid liquid separation which can be achieved by passive sedimentation (10% TS). Fresh faeces can therefore reduce the energy requirement for a thickening process when compared to wastewater, however, further research is required to investigate faecal material aging effects with respect to φ_g_ to understand the true penalty. Mechanical intervention to yield flow and compression is therefore only required at solids concentrations ≥10% TS. However, this can be managed by taking advantage of the thixotropic nature of fresh faeces. A τ_y_ reduction of more than 50% at 20% TS and up to 80% at 32% TS after 10 s at 10 s^−1^ was achieved. The introduction of an initial low shear period, could be integrated by the pumping process (i.e. screw pump, partial pump recycle or integrated macerator), which also advantages treatment by facilitating homogenisation for a uniform and predictable matrix. Such characteristics were also exhibited by SF, which behaved similarly from 5 to 35% TS and shear rates between 0.001 and 100 s^−1^. Yield stress and viscosity are accurately represented at 25% TS with a higher recovery of SF which will only overestimate energy needs, making it a suitable surrogate for RF. Therefore this study validates a safe, and consistent rheological substitute for the standardised development of faecal solids processes.

When transitioning from dewatering to drying processes, τ_s_ replaces τ_y_. At 25% TS, the greatest thixotropic breakdown occurs before faeces enter the sticky phase at 30–50% TS, where τ_y_ can be reduced by ∼70%–118 Pa and τ_s_ is ∼1000 Pa. It is therefore recommended that the dewatering phase commences at a solids concentration ≤25% TS and continues to 50% TS to bypass the sticky phase. Beyond the sticky phase, only τ_s_ applies, and faeces would yield by sliding under gravitational force. This would demand the lowest energy for material transfer to a drying process and dryer capacity would be maintained, which would otherwise be prone to faeces sticking if transferred before or during the sticky phase. However, the dewaterability of fresh faeces is not understood and is a research gap which is critically warranted for the development of low footprint solid/liquid separation. Alternatively, non-mechanical drying processes can be adopted such as drying beds to bypass the sticky phase (Strande et al., 2014), although requires a larger footprint.

This study has concluded that low energy and effective solid/liquid faecal sludge management is possible by decentralising separation at source, according to fresh faeces rheological properties. Decentralised treatment is gaining recognition as an economical alternative to centralised treatment in both low and high income countries where a sewerage network does not yet exist due to modular scalability and process flexibility (Eggimann et al., 2015; Jung et al., 2018). Understanding the flowability and stickiness of fresh faeces has highlighted the advantages and operational conditions for low energy solids phase treatment to provide confidence to uptake localised treatment, overcoming prejudices of decentralised treatment as more expensive and energetically costly due to economies of scale (Roefs et al., 2017). Further research is required to understand the rheological characteristics of faeces and paper as a composite material, to inform sanitation economics when paper is used for anal cleansing.

## Declaration of competing interest

The authors declare that they have no known competing financial interests or personal relationships that could have appeared to influence the work reported in this paper.

## Credit author statement

**Edwina Mercer:** Conceptualization; Methodology; Formal analysis; Validation; Investigation; Writing – original draft; Writing – review & editing; Project administration. **Shane P. Usher:** Conceptualization; Methodology; Formal analysis; Validation; Writing – original draft; Writing – review & editing. **Ewan J. McAdam:** Conceptualization; Methodology; Writing – original draft; Writing – review & editing; Supervision; Funding acquisition. **Brian Stoner:** Conceptualization; Writing – review & editing; Funding acquisition. **Yadira Bajón-Fernández:** Conceptualization; Methodology; Formal analysis; Validation; Writing – original draft; Writing – review & editing; Supervision; Project administration; Funding acquisition.
