# Supplementary material for: Rheological characterisation of synthetic and fresh faeces to inform on solids management strategies for non-sewered sanitation systems
Source: J Environ Manage. 2021 Dec 15;300:113730. doi: 10.1016/j.jenvman.2021.113730 (PMC8542804; doi:10.1016/j.jenvman.2021.113730)
Supplement: Multimedia component 1 [file mmc1.docx]

Supplementary data

Figure S1. Example of yield stress test using a vane method of 0.05 rpm at the vane radius for 300 seconds using real faeces at 25% total solids. Maximum shear stress value corresponds to shear yield stress.
