# Supplementary material for: Rheological characterisation of synthetic and fresh faeces to inform on solids management strategies for non-sewered sanitation systems
Source: J Environ Manage. 2021 Dec 15;300:113730. doi: 10.1016/j.jenvman.2021.113730 (PMC8542804; doi:10.1016/j.jenvman.2021.113730)
Supplement: Multimedia component 2 [file mmc2.docx]

Supplementary data

Figure S2. Example of structural recovery protocol for a 15% total solids real faeces sample. Pre-shear period of 30 seconds at 0.05 rpm (τ_y_ shear rate) to allow for the yield stress to be reached, 10 second shear period at 10 s^-1^ to initiate structural deformation, 360-second post-shear period (0.05 rpm) to allow for structural recovery.
