# Supplementary material for: Rheological characterisation of synthetic and fresh faeces to inform on solids management strategies for non-sewered sanitation systems
Source: J Environ Manage. 2021 Dec 15;300:113730. doi: 10.1016/j.jenvman.2021.113730 (PMC8542804; doi:10.1016/j.jenvman.2021.113730)
Supplement: Multimedia component 3 [file mmc3.docx]

Supplementary data

|  |
| --- |

Figure S3. Stool type characteristics according to total solids. N=40
